# Tradução para o português *An International Continence Society* (ICS) *report on the terminology for adult neurogenic lower urinary tract dysfunction* (ANLUTD)

**DOI:** 10.31744/einstein_journal/2022AE5680

**Published:** 2022-02-03

**Authors:** Ailton Fernandes, Carlos Alberto Ricetto Sacomani, Márcio Averbeck, José Antônio Prezotti, Ruiter Silva Ferreira, Daniel Moser, Jerzy B. Gajewski

**Affiliations:** 1 Hospital Universitário Pedro Ernesto Rio de Janeiro RJ Brasil Hospital Universitário Pedro Ernesto, Rio de Janeiro, RJ, Brasil.; 2 A.C. Camargo Cancer Center São Paulo SP Brasil A.C. Camargo Cancer Center, São Paulo, SP, Brasil.; 3 Hospital Moinhos de Ventos Porto Alegre RS Brasil Hospital Moinhos de Ventos, Porto Alegre, RS, Brasil.; 4 Hospital Santa Rita de Cássia Vitória ES Brasil Hospital Santa Rita de Cássia, Vitória, ES, Brasil.; 5 Hospital Estadual Alberto Rassi Goiânia GO Brasil Hospital Estadual Alberto Rassi, Goiânia, GO, Brasil.; 6 Instituto D’Or de Pesquisa e Ensino São Paulo SP Brasil Instituto D’Or de Pesquisa e Ensino, São Paulo, SP, Brasil.; 7 Dalhousie University Halifax Canadá Dalhousie University, Halifax, Canadá.

**Keywords:** Adulto, Disfunção, Neurogênica, Terminologia, Trato urinário inferior

## Abstract

**Introdução:**

A terminologia para disfunção neurogênica do trato urinário inferior em adultos (DNTUIA) deve ser definida e organizada com base clínica em um relatório de consenso.

**Métodos:**

Este relatório foi criado por um Grupo de Trabalho sob o endosso e diretrizes do *Standardization Steering Committee* (SSC) da *International Continence Society* (ICS), auxiliado em intervalos por julgadores externos. Todas as definições relevantes para DNTUIA foram atualizadas com base em pesquisas nos últimos 14 anos. Um extenso processo de 18 rodadas de revisão interna e externa foi realizado para examinar exaustivamente cada definição, com tomada de decisão pela opinião coletiva (consenso).

**Resultados:**

O Relatório de Terminologia para a DNTUIA, englobando 97 definições (42 novas e oito modificadas), foi desenvolvido. Este relatório é clinicamente baseado nos diagnósticos definidos mais comuns. Clareza e facilidade de uso têm sido os principais objetivos para torná-lo interpretável por profissionais e pessoas em treinamento em todos os diferentes grupos envolvidos não só na disfunção do trato urinário inferior, mas também em muitas outras especialidades médicas.

**Conclusão:**

Baseado no consenso, o Relatório de Terminologia para a DNTUIA foi produzido para auxiliar na pesquisa e na prática clínica.

## OBJETIVO DA TRADUÇÃO

O objetivo da tradução para a língua portuguesa é adequar e divulgar, de forma mais ampla, a terminologia atual padronizada pela *International Continence Society* (ICS) aos profissionais de saúde no Brasil.

## METODOLOGIA DA TRADUÇÃO

De acordo com o *Standardization Steering Committee* (SSC) da ICS, a tradução foi realizada seguindo as seguintes etapas:

- O escritório administrativo da ICS foi comunicado sobre o interesse da tradução do artigo por parte dos autores.
- O autor principal do documento e o SSC da ICS autorizaram a tradução e solicitaram a inclusão do autor principal no artigo traduzido.
- O documento em inglês foi traduzido para o português por um grupo de *experts* brasileiros (autores).
- A versão traduzida para o português foi retraduzida para o inglês por um autor independente.
- A versão retraduzida para o inglês foi submetida tanto ao autor principal do documento quanto ao SSC da ICS para revisão.
- Após correções realizadas, o documento traduzido para a língua portuguesa e o retraduzido para o inglês foram aprovados pelo autor principal e pelo SSC da ICS.

## 1 | INTRODUÇÃO

Este relatório é baseado na tradução para o português do relatório da *International Continence Society* (ICS) sobre a terminologia para disfunção neurogênica do trato urinário inferior em adultos (DNTUIA).^(1)^

“Adulto” refere-se a “um indivíduo totalmente crescido e fisicamente maduro”.^(2,3)^ “Neurogênico” refere-se à “origem do sistema nervoso”.^(3)^ “Trato Urinário Inferior (TUI)” refere-se à bexiga e à uretra (e à próstata, em homens).^(3)^ “Disfunção” refere-se à função anormal ou difícultada. “Disfunção neurogênica do trato urinário inferior em adultos” refere-se à função anormal ou difícil da bexiga e da uretra (e/ou da próstata em homens) em indivíduos maduros no contexto de uma doença neurológica significativa clinicamente confirmada. Atualmente, não há um único documento focalizando as definições relacionadas à DNTUIA. Muitos sinais e sintomas de DNTUIA foram definidos nos atuais relatórios de terminologia para disfunções do TUI e do assoalho pélvico.^(4-6)^ Com a vantagem de pesquisas em andamento sobre a epidemiologia, a fisiopatologia e as iniciativas farmacológicas de médicos generalistas e especialistas em DNTUIA, é oportuno reconsiderar as diferentes definições.

## 2 | METODOLOGIA

Este documento foi desenvolvido de acordo com a metodologia publicada pelo *Standardization Steering Committee* da *International Continence Society*.^(7)^ Este documento está alinhado com as padronizações anteriores da ICS em disfunção do TUI e adaptado para um grupo de pacientes com DNTUIA. Assim, a DNTUIA pode ser diagnosticada somente na presença de doença neurológica. A intenção é substituir a terminologia antiga de “bexiga neurogênica” ou “disfunção neurogênica da bexiga”: essas definições são enganosas, porque a(s) disfunção(ões) pode envolver não apenas a bexiga, mas também a competência ou o relaxamento do esfíncter uretral. Além disso, usar um único termo para indicar um amplo espectro de disfunções é restritivo e pouco claro. Por exemplo, existem muitas diferenças, em termos de investigações necessárias, tratamento e prognóstico, entre um paciente masculino com lesão medular em nível cervical e uma paciente com doença de Parkinson, ambos com queixa de sintomas do trato urinário inferior (STUI) e “rotulados” como tendo uma “bexiga neurogênica”. Finalmente, essas definições podem levar à convicção de que a disfunção pode ser causada por um problema na bexiga, enquanto o defeito primário está no sistema nervoso central ou periférico. O documento contém algumas padronizações originais de definições relacionadas aos STUI, algumas modificadas com a designação “MODIFICADO” e algumas recém-definidas “NOVO”.

Este Relatório de Terminologia é inerentemente e apropriadamente um documento de definição, reunindo as definições desses termos, isto é, “palavras usadas para expressar um conceito definido em um ramo particular de estudo”,^(8)^ aqui DNTUIA. A ênfase tem sido abrangente, incluindo os termos atualmente em uso na literatura pertinente revisada por pares. As definições desses termos serão revisadas com todas as evidências disponíveis. O objetivo é auxiliar a pesquisa e a prática. Alguns termos novos e revisados foram incluídos. Notas explicativas sobre definições foram referidas, quando possível, à seção “Notas de rodapé”. Como todos os outros relatórios conjuntos de terminologia da ICS, todos os esforços foram feitos para garantir que este relatório seja:

1. Fácil de usar: deve ser entendido por todos os clínicos e pesquisadores.
2. Baseado na clínica: refere-se à prática clínica relevante.
3. Origem: quando a definição existente de um termo (de uma das múltiplas fontes utilizadas) for considerada apropriada, essa definição permanecerá e será devidamente referenciada. Um grande número destas, devido ao seu uso a longo prazo, tornou-se agora genérico, como é evidente por sua listagem em dicionários médicos.
4. Capaz de fornecer explicações: quando uma explicação específica for considerada apropriada para descrever uma alteração de definições anteriores ou para qualificar a definição atual, ela será incluída como nota de rodapé deste documento. Sempre que possível, os princípios médicos baseados em evidências serão seguidos.

Este documento envolveu 18 rodadas de revisão completa, por coautores, de um rascunho inicial (Versão 1) concluído em 16 de setembro de 2014. Os comentários para cada rodada de revisão foram compilados e debatidos, conforme necessário, para formar uma versão subsequente. Encontros presenciais sobre o documento foram realizados em Zurique e Tóquio.*

Este documento abrange sinais, sintomas, observações e definições urodinâmicas, diagnósticos clínicos e tratamento.

## 3 | RESULTADOS

### 1. Sintomas da disfunção neurogênica do trato urinário inferior

Sintoma: qualquer fenômeno mórbido ou desvio do normal em estrutura, função ou sensibilidade, experimentado pelo indivíduo e indicativo de doença ou problema de saúde.^(3)^ Os sintomas podem ser relatados espontaneamente ou obtidos por meio do paciente, do cuidador ou de voluntários.^(4-6)^ Os STUI são classificados como neurogênicos SOMENTE na presença de uma doença neurológica significativa. Os sintomas são um indicador subjetivo de mudança na doença como percebido pelo paciente, cuidador ou parceiro, que pode levar o paciente a procurar ajuda de profissionais de saúde. Eles são geralmente qualitativos. Em geral, STUI não pode ser usado para fazer um diagnóstico definitivo. Sintomas do trato urinário inferior em pessoas com doença neurológica também podem indicar outras patologias, além da DNTUIA, como infecção urinária.

Três grupos de STUI são: sintomas de armazenamento, esvaziamento e pós-miccionais.

**1.1. Sintomas de armazenamento:** ocorrem durante a fase de armazenamento da bexiga (MODIFICADO).^†^

**1.1.1. Aumento da frequência urinária diurna:** queixa de que a micção ocorre com maior frequência durante as horas de vigília do que anteriormente considerado normal.^(6)^

**1.1.2. Noctúria:** é a queixa de acordar para urinar durante o período de sono principal^(9)^ (MODIFICADO).

**1.1.3. Urgência:** é a queixa de um desejo súbito de urinar, o que é difícil postergar.

**1.1.4. Incontinência urinária:** queixa de perda involuntária de urina.^(6)^

**1.1.4.1. Incontinência urinária de esforço:** é a queixa de perda involuntária por esforço, ou por espirro ou por tosse.^(5)^

**1.1.4.2. Incontinência urinária de urgência:** é a queixa de perda involuntária de urina associada à urgência.^(6)^

**1.1.4.3. Incontinência urinária mista:** é a queixa de perda involuntária associada à urgência e também a esforço, espirro ou tosse.^(6)^

**1.1.4.4. Enurese:** queixa de incontinência intermitente que ocorre durante períodos de sono^(9)^ (NOVO).

**1.1.4.4.1. Enurese primária:** presente desde o nascimento (NOVO).

**1.1.4.4.2. Enurese adquirida:** é uma enurese desenvolvida na vida adulta (NOVO).*

**1.1.4.5. Incontinência (urinária) contínua:** queixa de perda involuntária contínua de urina.^(6)^

**1.1.4.6. Incontinência urinária por *deficit* cognitivo:** queixa de incontinência urinária periódica que o indivíduo com comprometimento cognitivo relata ter ocorrido sem estar ciente (NOVO).

**1.1.4.7. Incontinência urinária por dificudades de mobilidade:** queixa de incapacidade de chegar a tempo ao vaso sanitário para a micção, devido à incapacidade física ou médica (NOVO).^||^

**1.1.4.8. Incontinência urinária da atividade sexual** é o relato individual de incontinência urinária durante ou associada à atividade sexual (NOVO).^¶^

**1.1.4.9. Outros tipos situacionais de incontinência urinária:** podem existir, como, por exemplo, incontinência do riso ou incontinência associada a crises epilépticas e à denervação esfincteriana da cauda equina e nas lesões do núcleo de Onuf, na atrofia de múltiplos sistemas (NOVO).

**1.1.5. Sensibilidade vesical:** pode ser definida, durante a anamnese, seguindo as categorias.

**1.1.5.1. Normal:** o indivíduo está consciente do enchimento da bexiga e a sensibilidade aumenta até um forte desejo para urinar.^(5)^

**1.1.5.2. Sensibilidade vesical aumentada:** queixa de que o desejo de urinar durante o enchimento da bexiga ocorre mais cedo ou é mais persistente do que o experimentado anteriormente. Isso difere da urgência, pelo fato de que a micção pode ser adiada, apesar do desejo de urinar.^(6)^

**1.1.5.3. Sensibilidade vesical reduzida:** queixa de que o desejo definitivo de urinar ocorre mais tarde àquele previamente experimentado, apesar da consciência de que a bexiga está enchendo.^(6)^

**1.1.5.4. Ausente:** o indivíduo não relata sensibilidade de enchimento da bexiga ou desejo de urinar.^(5)^

**1.1.5.5. Consciência de sensibilidades não específicas da bexiga:** o indivíduo não relata nenhuma sensibilidade específica da bexiga, mas pode perceber, por exemplo, plenitude abdominal, sintomas vegetativos, espasticidade ou sensibilidades uretrais, como consciência de enchimento da bexiga ou sinal de plenitude vesical (MODIFICADO).

**1.1.5.6. Sensibilidades anormais:** consciência sobre a sensibilidade na bexiga, uretra ou pelve, descrita com palavras como “formigamento”, “queimação” ou “choque”, no contexto de uma doença neurológica clinicamente significativa (exemplo: lesão incompleta da medula espinhal) (NOVO).

**1.1.5.7. Dor na bexiga:** queixa de dor supra ou retropúbica, pressão ou desconforto, relacionados à bexiga e, usualmente, aumentando com o enchimento vesical. A dor pode persistir ou aliviar após a micção.^(5)^

**1.2. Sintomas de esvaziamento:** algo distinto da sensibilidade ou função normal, que acomete uma pessoa durante o ato miccional.^(3)^

**1.2.1. Jato fraco:** queixa de um jato urinário mais fraco em comparação ao desempenho prévio do paciente ou em comparação com outros indivíduos.^(6)^

**1.2.2. Jato urinário dividido ou espalhado:** queixa de que a passagem da urina ocorre em jato dividido ou espalhado, ao invés de um jato único discreto.^(6)^

**1.2.3. Jato intermitente (intermitência):** é o termo utilizado quando o indivíduo descreve um jato de urina que para e recomeça em uma ou mais ocasiões durante a micção.^(5)^

**1.2.4. Hesitação:** queixa de atraso no início da micção.^(6)^

**1.2.5. Esforço para urinar:** queixa de necessidade de fazer esforço intenso (por meio de contração abdominal, Valsalva ou pressão suprapúbica) para começar, manter ou melhorar o jato urinário.^(6)^

**1.2.6. Gotejamento terminal:** é o termo utilizado quando um indivíduo descreve uma fase final da micção prolongada, na qual o fluxo se torna reduzido a pingos ou gotejamento.^(5)^

**1.3. Sintomas pós-miccionais:** são relatados imediatamente após a micção.^(5)^

**1.3.1. Sensibilidade de esvaziamento incompleto:** queixa de que a bexiga, após a micção, não parece estar vazia.^(6)^

**1.3.2. Perda pós-miccional:** queixa de perda involuntária de urina, que ocorre após o final da micção.^(5,6)^

### 2. SINAIS DA DISFUNÇÃO NEUROGÊNICA DO TRATO URINÁRIO INFERIOR

**Sinal:** qualquer anormalidade indicativa de doença ou de um problema de saúde detectada durante o exame do paciente; um indicativo objetivo de doença^(3)^ ou de um problema de saúde. Os sinais são observados pelo médico, incluindo maneiras simples de verificar e quantificar os sintomas.

A mensuração da frequência, gravidade e impacto dos STUI solicitando ao paciente que registre micções e sintomas por um período de dias fornece informações valiosas. O registro de “eventos miccionais” pode ser feito de três formas principais.^**^

**2.1. Quadro de horário miccional:** registro simples do horário das micções, durante o dia e a noite, por, no mínimo, 24 horas.^(5)^

**2.2. Registro de frequência e volume (RFV):** registro do volume urinado, bem como do horário de cada micção, durante o dia e a noite, por, no mínimo, 24 horas.^(5)^

**2.3. Diário miccional:** registro do horário das micções e dos volumes urinados, episódios de incontinência, uso de absorventes e outras informações, como o volume de líquidos ingerido, o grau de urgência e o grau de incontinência.^(5,10)^††

### 3. ACHADOS E DEFINIÇÕES URODINÂMICOS DA DNTUIA

**3.1. Definições da cistometria**

A função de armazenamento da bexiga deveria ser descrita de acordo com a sensibilidade da bexiga, a atividade detrusora, a complacência vesical e a capacidade vesical. Anormalidades identificadas no armazenamento podem ou não ser o resultado de uma doença neurológica clinicamente significativa.

**3.1.1. Sensibilidade vesical durante a cistometria**

**3.1.1.1. Sensibilidade vesical normal:** pode ser avaliada por três pontos definidos (de acordo com as recomendações da ICS) durante a cistometria: primeira sensibilidade de enchimento vesical, primeiro desejo miccional e forte desejo miccional, os quais são avaliados em relação ao volume vesical naquele momento e em relação às queixas de sintomas do paciente.^(5,8)^

**3.1.1.2. Sensibilidade vesical reduzida:** percepção de sensibilidade vesical reduzida durante a cistometria.^(6)^

**3.1.1.3. Sensibilidade vesical ausente:** o paciente não refere sensibilidade vesical durante a cistometria.^(6)^

**3.1.1.4. Sensibilidade vesical aumentada:** percepção de sensibilidade vesical aumentada durante a cistometria com primeiro desejo miccional precoce; forte desejo miccional precoce, o qual ocorre com baixo volume vesical, e capacidade cistométrica máxima reduzida e sem aumento anormal da pressão detrusora.^(6)^

**3.1.1.5. Sensibilidade anormal:** percepção de sensibilidade na bexiga, uretra ou pelve, descrita com palavras como “formigamento”, “queimação” ou “choque”, no contexto de doença neurológica clinicamente significativa (exemplo: lesão de medula espinhal incompleta) (NOVO).

**3.1.1.6. Percepção vesical não específica:** percepção do enchimento da bexiga como plenitude abdominal, sintomas vegetativos, espasticidade ou outras “sensibilidade não específicas”, no contexto de doença neurológica clinicamente significativa (exemplo: lesão de medula espinhal incompleta) (NOVO).

**3.1.1.7. Dor vesical:** uma sensibilidade desconfortável (dor, pressão e desconforto) cuja percepção é relacionada à bexiga. bexiga (MODIFICADO).^‡‡^

**3.1.2. Capacidade vesical durante a cistometria**

**3.1.2.1. Capacidade cistométrica:** é o volume da bexiga no final da cistometria, quando é dada a “permissão para urinar ou para esvaziar a bexiga”. O ponto exato deve ser especificado, por exemplo: se a infusão é interrompida quando o paciente tem um desejo normal de urinar. A capacidade cistométrica é o volume urinado somado com o residual.^(5)^

**3.1.3. Função detrusora durante a cistometria**

**3.1.3.1. Hiperatividade detrusora neurogênica:** é uma observação urodinâmica caracterizada por contrações involuntárias do detrusor durante a fase de enchimento, as quais podem ser espontâneas ou provocadas, no contexto de doença neurológica clinicamente significativa.^(5)^

Tipos específicos de hiperatividade detrusora neurogênica incluem:

**3.1.3.1.1. Hiperatividade detrusora fásica** é definida por uma forma de onda característica, podendo ou não levar à incontinência urinária.^(5)^

**3.1.3.1.2. Hiperatividade detrusora terminal** é definida como uma contração involuntária do detrusor ocorrendo na capacidade cistométrica máxima ou próximo dela, a qual não pode ser inibida, resultando em incontinência ou, mesmo, esvaziamento vesical reflexo (micção reflexa) (MODIFICADO).^##^

**3.1.3.1.3. Hiperatividade detrusora sustentada** é definida como uma contração detrusora contínua sem retorno à pressão detrusora basal (NOVO).

**3.1.3.1.4. Hiperatividade detrusora composta** é definida como uma contração detrusora fásica seguida de um aumento da pressão detrusora e pressão de base a cada contração subsequente (NOVO).

**3.1.3.1.5. Hiperatividade detrusora de alta pressão** é definida como a máxima hiperatividade detrusora fásica, terminal, sustentada ou composta, com a máxima pressão detrusora interpretada pelo investigador como potencialmente prejudicial à função renal e/ou a saúde do paciente, sendo que o valor deve ser definido no laudo (NOVO).

**3.1.3.1.6. Incontinência por hiperatividade detrusora neurogênica** é a incontinência causada por hiperatividade detrusora neurogênica involuntária (NOVO).***

**3.1.3.2. Pressões de perda**

**3.1.3.2.1. Pressão de perda do detrusor (PPD):** é definida como a pressão detrusora mais baixa em que ocorre a perda de urina na ausência de uma contração detrusora ou aumento da pressão abdominal.

**3.1.3.2.2. Pressão de perda por hiperatividade detrusora (PPHD):** é definida como a menor elevação da pressão detrusora em que ocorre a primeira perda de urina com a hiperatividade detrusora na ausência de contração voluntária do detrusor ou aumento da pressão abdominal (NOVO).

**3.1.3.2.3. Volume na perda detrusora (VPD):** é definido como o volume vesical em que ocorre a primeira perda de urina, com hiperatividade detrusora ou baixa complacência (NOVO).

**3.1.3.2.4. Pressão de perda abdominal (PPA):** é a pressão intravesical em que ocorre a perda de urina devido ao aumento da pressão abdominal na ausência de uma contração detrusora.^(5)^

**3.1.3.2.5. Complacência vesical:** descreve a relação entre as mudanças no volume e na pressão detrusora durante a cistometria.^(5)^

**3.2. Definições do estudo fluxo pressão**

**3.2.1. Função detrusora, durante a fase miccional, em pessoas que podem iniciar micção voluntária.**

**3.2.1.1. Função detrusora normal:** é uma contração detrusora contínua iniciada voluntariamente que leva ao completo esvaziamento vesical, dentro de um período de tempo normal e na ausência de obstrução. Para uma dada contração detrusora, a magnitude da elevação da pressão reportada dependerá do grau de resistência infravesical.^(5)^

**3.2.1.2. Hipoatividade detrusora neurogênica:** é definida como uma contração de duração e/ou força reduzida, resultando em um esvaziamento vesical prolongado e/ou incapacidade de obter esvaziamento vesical completo dentro de um período de tempo normal, no cenário de uma doença neurológica clinicamente significante (NOVO).

**3.2.1.3. Acontratilidade detrusora neurogênica:** é aquela contração que não pode ser demonstrada durante os estudos urodinâmicos no cenário de uma lesão neurológica clinicamente significante (NOVO).

**3.2.1.4. Esvaziamento vesical balanceado:** é um esvaziamento vesical com baixo resíduo e pressão detrusora fisiológica, percebido pelo investigador e que deve ser relatado no laudo (NOVO).

**3.2.2**. Função detrusora durante estudos fluxo pressão em pessoas que não podem iniciar micção voluntária.

**3.2.2.1. Esvaziamento vesical iniciado por reflexo:** é entendido como um reflexo do TUI obtido artificialmente por meio de várias manobras (estímulos exógenos) realizadas pelo paciente ou terapeuta, resultando em esvaziamento vesical completo ou incompleto (NOVO).^§§§^

**3.2.3.** Função do esfíncter durante estudos fluxo pressão.

**3.2.3.1. Dissinergia detrusor-esfincteriana (DDE):** descreve uma contração involuntária do esfíncter estriado uretral e/ou periuretral concomitante com uma contração detrusora. Ocasionalmente, o fluxo pode ser completamente impedido.^(5)^

**3.2.3.2. Ausência de relaxamento do esfíncter uretral:** é caracterizado por um esfíncter uretral que não relaxa, resultando em obstrução e fluxo urinário reduzido.^(5)^

**3.2.3.3. Relaxamento tardio do esfíncter uretral:** é caracterizado pelo relaxamento insuficiente e inadequado do esfíncter durante tentativa de micção, resultando em atraso do fluxo de urina (NOVO).^###^

### 4. DISFUNÇÃO NEUROGÊNICA DO TRATO URINÁRIO INFERIOR – DIAGNÓSTICO CLÍNICO

Diagnósticos clínicos são manifestações dos sinais e sintomas caracterizados por achados específicos e/ou evidências não urodinâmicas, definidas pela presença de observações urodinâmicas associadas com sinais ou sintomas característicos e/ou evidência não urodinâmica de processo patológico relevante.

Isso depende da extensão da perda da função neurológica e de qual parte do sistema nervoso é afetada. Lesões neurais são descritas de acordo com o tempo de início, nível neurológico, integridade e risco de progressão.

**4.1. Fase de choque medular:** é geralmente temporária, após um trauma raquimedular ou lesão neurológica aguda, caracterizada por perda da atividade motora, sensitiva e reflexa, abaixo do nível da lesão. Disfunção neurogênica do TUI no choque medular: caracteriza-se, geralmente, por uma retenção urinária temporária, completamente sem dor (NOVO).

**4.2. Lesão medular suprapontina (LMSP):** é uma lesão neurológica acima da ponte (prosencéfalo ou mesencéfalo). Disfunção neurogênica do TUI na LMSP: há uma contração detrusora reflexa, com regulação cerebral e inibição central inadequadas, e, geralmente, esvaziamento vesical sinérgico (NOVO).^****^

**4.3. Lesão medular suprassacral/lesão pontina (LMSS):** é uma lesão neurológica na medula suprassacral e/ou ponte. Disfunção neurogênica do TUI na LMSS: hiperatividade detrusora (HD) e incontinência por HD são comuns, com ou sem dissinergia detrusor-esfíncter (DDE) uretral, frequentemente resultando em bexiga de “alta pressão” e resíduo pós-miccional significativo (NOVO).^††††^

**4.4. Lesão medular sacral (LMS):** é uma lesão neurológica na medula espinal sacral. Disfunção neurogênica do TUI na LMS: os achados incluem detrusor acontrátil, com ou sem redução da complacência vesical e, geralmente, com atividade esfincteriana comprometida (NOVO).^‡‡‡‡^

**4.5. Lesão infrassacral (cauda equina e nervos periféricos) (LCENP):** é uma lesão neurológica que afeta a cauda equina e/ou nervos periféricos. Disfunção neurogênica do TUI em LCENP: acontratilidade detrusora e/ou incontinência urinária de esforço (IUE) podem estar presentes. Na neuropatia diabética, hiperatividade detrusora pode ser encontrada em combinação com as anteriores (NOVO).^§§§§^

**Lesão neuronal mista:** é resultante de lesão simultânea das vias neurais em diferentes níveis do sistema nervoso central (NOVO).

**Disreflexia autonômica:** é uma síndrome resultante da lesão medular cervical ou torácica superior, acima de T6, desencadeada por estímulos na região de distribuição do núcleo autônomo do simpático, caracterizada por uma função simpática não regulada abaixo do nível da lesão e resposta autonômica compensatória (NOVO).^||||||||^

**Disreflexia autonômica assintomática:** aumento da pressão arterial sem nenhum outro sintoma (NOVO).^¶¶¶¶^

**Bexiga hiperativa neurogênica:** é caracterizada por urgência, com ou sem incontinência urinária de urgência, geralmente com aumento da frequência diurna e noctúria na vigência de uma doença neurológica clinicamente significante com, pelo menos, a sensibilidade parcialmente preservada (NOVO).^####^

**Desregulação miccional:** é o ato de urinar em situações geralmente consideradas socialmente inadequadas, como ainda estar totalmente vestido ou em ambiente público, longe de instalações sanitárias (NOVO).

**Micção involuntária:** é um sintoma e um diagnóstico de esvaziamento vesical esporádico quando se está acordado, porém sem intenção de urinar (NOVO).^*****^

**Retenção urinária:** é a incapacidade de esvaziar a bexiga adequadamente e pode ser dividida em aguda, crônica, completa e incompleta (NOVO).

**Retenção urinária aguda:** é definida como um evento agudo doloroso, quando o paciente tem a bexiga cheia, palpável ou percutível e é incapaz de eliminar qualquer volume de urina.^(6)^

**Retenção urinária crônica:** é definida como bexiga sem dor que permanece palpável ou percutível após o paciente ter urinado. Esses pacientes podem ser incontinentes.^(5)^

**Retenção urinária completa:** é a incapacidade de esvaziar qualquer quantidade do volume da bexiga ou a necessidade de uso de um cateter, consciente ou inconscientemente, devido à obstrução infravesical anatômica ou funcional, hipoatividade do detrusor ou ambas (NOVO).

**Retenção urinária incompleta:** é o esvaziamento vesical inadequado, devido à obstrução infravesical anatômica ou funcional, à hipoatividade do detrusor ou por ambas, quando o volume urinado é menor que o resíduo pós-miccional.

**Resíduo pós-miccional:** é definido como o volume de urina deixado na bexiga ao final da micção.^(5)^

### 5. DEFINIÇÕES DE TRATAMENTO

**5.1. Ativadores do reflexo vesical:** compreende várias manobras realizadas pelo paciente ou terapeuta para provocar o reflexo de esvaziamento por estímulos exteroceptivos (relacionado a ou ativado por estímulos recebidos de fora da bexiga.^(5)^

**5.2. Compressão da bexiga:** refere-se a várias manobras de compressão destinadas a aumentar a pressão intravesical para facilitar o esvaziando da bexiga, com ou sem uma clara sensibilidade vesical (MODIFICADO).^||||||||||^

**5.3. Cateterismo:** é uma técnica para esvaziamento da bexiga empregando um cateter para drená-la ou um reservatório urinário.^(5)^

**5.3.1. Cateterismo de demora:** um cateter, reservatório urinário ou conduto urinário permanece na bexiga, durante período superior a um esvaziamento.^(5)^

**5.3.2. Cateterismo intermitente:** é definido como a drenagem da bexiga ou de um reservatório urinário com subsequente remoção do cateter, geralmente em intervalos regulares (MODIFICADO).

**5.3.2.1. Cateterismo intermitente limpo:** utiliza uma técnica limpa. Isso implica em técnicas regulares de higiene das mãos e genitália e no uso de cateteres reutilizáveis ou descartáveis (MODIFICADO).

**5.3.2.2. Cateterismo intermitente asséptico:** isso implica em preparação antisséptica da genitália e uso de cateteres estéreis (uso único) e instrumentos/luvas em uma área limpa designada (NOVO).

**5.3.2.3. Cateterismo intermitente estéril:** configuração completamente estéril, incluindo antissepsia da pele e genitália, luvas estéreis, pinças, avental e máscara (NOVO).^#####^

**5.3.2.4. Cateterismo intermitente técnica sem contato:** foi introduzido como uma maneira mais fácil para o paciente realizar autocateterismo intermitente com um cateter pronto para uso (cateter pré-lubrificado, geralmente hidrofílico). Uma área para puxar ou embalagens especiais são utilizadas para manipulá-lo, sem tocar diretamente em sua superfície de deslizamento (NOVO).^******^

**5.4. Eletroestimulação**

**5.4.1. Neuroestimulação elétrica direta:** é uma estimulação direta dos nervos ou tecido neural para efetuar (desencadear) a função do órgão final. Isso é realizado por meio de eletrodos implantados diretamente ou próximo do nervo ou tecido neural (NOVO).^††††††^

**5.4.2. Neuromodulação elétrica:** é a estimulação dos nervos ou tecido neural para modular a função induzir resposta terapêutica do TUI (NOVO).^‡‡‡‡‡‡^

**5.4.3. Estimulação elétrica transcutânea do nervo:** é a estimulação elétrica dos nervos por meio da pele intacta, para modular função e induzir resposta terapêutica do TUI (NOVO).^§§§§§§^

**5.4.4. Estimulação elétrica pélvica:** é aplicação da corrente elétrica para estimular as vísceras pélvicas ou seu suprimento nervoso (NOVO).^||||||||||||^

## 4 | CONCLUSÃO

Terminologia padronizada é um aspecto importante na pesquisa e comunicação na disfunção neurogênica do trato urinário inferior. A *International Continence Society* continua a ter um papel fundamental na padronização da terminologia relacionada ao trato urinário inferior e disfunção de órgãos pélvicos.
